# Catastrophizing questionnaire: translation and psychometric validation in Brazilian adult orthodontic patients

**DOI:** 10.1590/2177-6709.31.2.e2625245.oar

**Published:** 2026-06-22

**Authors:** Tony Vieira FARIA, Luiz Gonzaga GANDINI, Lucas Arrais CAMPOS

**Affiliations:** 1 São Paulo State University, Dental School (Araraquara/SP, Brazil).; 2 University of Eastern Finland, Institute of Dentistry, Faculty of Health Sciences (Kuopio, Finland).

**Keywords:** Psychometrics, Catastrophization, Validation study, Orthodontics, Psicometria, Catastrofização, Estudos de validação, Ortodontia

## Abstract

**Objective:**

This study aimed to translate, culturally adapt, and estimate the psychometric properties of the Catastrophizing Questionnaire (CQ) for use in Portuguese among orthodontic patients.

**Methods:**

The translation process followed international guidelines, including forward and backward translation. Confirmatory factor analysis (CFA) was conducted to assess factorial validity, using indices such as the Comparative Fit Index (CFI), Tucker-Lewis Index (TLI), Root Mean Square Error of Approximation (RMSEA), and Standardized Root Mean Square Residual (SRMR). Convergent validity [Average Variance Extracted (AVE)] and reliability were estimated.

**Results:**

A total of 334 Brazilian orthodontic patients (74.6% female, mean age = 30.6 years, standard deviation 12.4) participated in the study. The factor model of the Portuguese version of CQ presented an adequate fit to the data (CFA: CFI = 0.95, TLI = 0.94, RMSEA = 0.07, and SRMR = 0.06). Reliability was acceptable (α and ω ≥ 0.93). Although the AVE was slightly below the recommended threshold (AVE = 0.41), the overall model fit and internal consistency supported the adequacy of convergent validity.

**Conclusion:**

The Portuguese CQ showed adequate psychometric properties for assessing catastrophizing in Brazilian adult orthodontic patients. As a pain-independent measure, it enables the early identification of catastrophizing tendencies before appliance placement, supporting a patient-centered approach. By helping clinicians recognize individual psychosocial vulnerabilities, the CQ can guide tailored communication and management strategies aimed at improving the overall treatment experience.

## RESUMO

**Objetivo:**

Este estudo teve como objetivo traduzir, adaptar culturalmente e estimar as propriedades psicométricas do Questionário de Catastrofização (QC) para uso em português em pacientes ortodônticos.

**Métodos:**

O processo de tradução seguiu diretrizes internacionais, incluindo tradução direta e retrotradução. Foi realizada análise fatorial confirmatória (AFC) para avaliar a validade fatorial, utilizando índices como o *Comparative Fit Index* (CFI), *Tucker-Lewis Index* (TLI), *Root Mean Square Error of Approximation* (RMSEA) e *Standardized Root Mean Square Residual* (SRMR). A validade convergente [Variância Extraída Média (VEM)] e a confiabilidade foram estimadas.

**Resultados:**

Participaram do estudo 334 pacientes ortodônticos brasileiros (74,6% do sexo feminino, idade média = 30,6 anos, desvio-padrão = 12,4). O modelo fatorial da versão em português do QC apresentou ajuste adequado aos dados (AFC: CFI = 0,95, TLI = 0,94, RMSEA = 0,07 e SRMR = 0,06). A confiabilidade foi adequada (α e ω ≥ 0,93). Apesar da VEM ter apresentado um valor marginalmente inferior ao ponto de corte recomendado (VEM = 0,41), o ajuste geral do modelo e a consistência interna corroboraram a adequação da validade convergente.

**Conclusões:**

A versão portuguesa do QC demonstrou propriedades psicométricas adequadas para avaliar traços de catastrofização em adultos brasileiros submetidos a tratamento ortodôntico. Por se tratar de uma medida independente da dor, o QC possibilita a identificação precoce de tendências ao catastrofismo antes da instalação do aparelho ortodôntico, favorecendo uma abordagem centrada no paciente. Ao auxiliar os clínicos a reconhecer vulnerabilidades psicossociais individuais, o QC pode orientar estratégias de comunicação e manejo personalizadas, com o objetivo de aprimorar a experiência geral do tratamento.

## INTRODUCTION

A catastrophizing patient anticipates and interprets any negative experience as catastrophic and intolerable, often perceiving it as more severe in anticipation than it is.^1^ Catastrophizing involves persistent concern that negative events, perceived as catastrophes, will recur.^2^ It also involves a subjective perception that exaggerates and amplifies the severity of such events.^2^ A further component is a sense of helplessness and the perceived lack of effective coping strategies.^3^ ^,^ ^4^ Together, they represent the core cognitive and emotional components of the catastrophizing construct and are referred to as Rumination, Magnification, and Helplessness, respectively.^5^

In Dentistry, catastrophic thinking is associated with anticipatory and treatment-related dental anxiety. This suggests a shared cognitive and neurophysiological basis between anxiety and catastrophizing.^6^ Such an excessive focus on negative health-related events may lead individuals to avoid seeking dental care.^3^ In orthodontic treatment specifically, assessing catastrophizing prior to treatment may be clinically relevant. It may influence patient adherence, anxiety levels, subjective experience of orthodontic pain, and potentially contribute to treatment discontinuation.^3^ ^,^ ^7^ ^,^ ^8^

Catastrophizing is a subjective construct and can be indirectly measured through psychometric scales. Most established conceptual and measurement approaches have been grounded in pain-related experiences. The Pain Catastrophizing Scale (PCS)^4^ is among the most widely recognized and extensively used instruments. Responses are based on thoughts and feelings related to the experience of pain.^3^ ^,^ ^4^ Therefore, respondents typically complete this scale while in pain or by recalling previous painful experiences. Consequently, in orthodontics, the PCS is primarily informative after orthodontic pain begins, that is, following appliance placement and activation.^7^ Although assessment after pain onset has clinical value, it is also important to identify patients predisposed to catastrophizing before treatment begins. It may help orthodontists to anticipate maladaptive responses to orthodontic treatment and support a more tailored approach to managing orthodontic patient expectations and behavior.^9^

The Catastrophizing Questionnaire (CQ)^2^ is an innovative approach because, in contrast to pain-focused instruments such as the PCS, it assesses catastrophizing as a general cognitive trait independent of current pain context. This distinction is relevant in Orthodontics, as it allows for the identification of psychological vulnerabilities before treatment begins. The CQ was originally developed in English in the United Kingdom. The development sample included fluent English speakers from the general population and individuals with self-reported histories of mental health conditions.^2^ In this sample, the CQ showed a unidimensional structure and adequate psychometric properties for identifying a catastrophizing profile before the onset of pain.^2^ These features suggest its potential applicability to patients undergoing orthodontic treatment. Therefore, translating and culturally adapting the CQ to other languages and populations may contribute to both clinical practice and research, enabling a patient-centered approach.

Although psychometric scales like CQ are valuable tools for this purpose, relying solely on raw scores may oversimplify the latent construct. It may also compromise the validity and reliability of the data. When a scale is applied to new populations, its psychometric properties must be evaluated to confirm data validity and reliability and support score interpretation in the new context.^9^ ^,^ ^10^ In psychometric analyses, participants’ responses to each item are treated as observed (manifest) variables. In contrast, the construct of interest (e.g., catastrophizing) is conceptualized as a latent variable. Latent variables are not directly observable; they are modeled and inferred from the shared variance among the observed variables.^11^

Despite the clinical relevance of catastrophizing in Orthodontics, there are currently no Portuguese scales with established psychometric properties to measure this cognitive trait independently of pain, especially before orthodontic treatment begins. Therefore, the aims of the present study were to translate and culturally adapt the CQ to Brazilian Portuguese and to estimate its psychometric properties when applied to Brazilian adults undergoing orthodontic treatment.

## MATERIAL AND METHODS

### STUDY DESIGN AND SAMPLING

This was a cross-sectional study with a non-probabilistic (convenience) sampling, which is a common approach in psychometric validation research.^10^ ^,^ ^12^ ^-^ ^17^ The sample was composed of Brazilian orthodontic patients aged 18 years or older, residing in the state of Espírito Santo. Participants were initially recruited from the Brazilian Association of Dentistry - Espírito Santo Section (ABO-ES, Brazil), during ongoing orthodontic treatment with either fixed appliances or clear aligners, as part of a postgraduate specialization course in Orthodontics.

The minimum required sample size was calculated following the proposal of Hair et al.^18^, who recommend a minimum of 10 respondents per observed variable (item) of the factor model. The CQ includes 24 items, therefore, the minimum sample size was 240 participants. Accounting for a potential loss rate of 20%, the minimum sample size for the study was increased to 300 individuals.

### ETHICAL ASPECTS AND PROCEDURES

This study was approved by the Research Ethics Committee of the Araraquara School of Dentistry (protocol number CAAE 74740923.3.0000.5416 and conducted in full accordance with ethical principles, including the World Medical Association Declaration of Helsinki.^19^ All participants provided written informed consent. Data collection was conducted in person at the clinic, from June to September 2024, prior to clinical appointments, in a private room designated for this purpose. The questionnaire was self-administered digitally using LimeSurvey software (LimeSurvey GmbH, Hamburg, Germany; http://www.limesurvey.org).

### SAMPLE CHARACTERISTICS

Demographic data were collected for sample characterization, including age (in full years), sex (male or female), marital status (single; married or in a stable union; divorced or separated; widowed), educational level (incomplete elementary; completed elementary; completed high school or incomplete higher education; completed higher education), and monthly household income. In the present study, demographic variables were used exclusively to describe the sample and were not incorporated as predictors in the subsequent statistical analyses.

### MEASUREMENT INSTRUMENTS

This study employed two instruments: the Catastrophizing Questionnaire (CQ, Supplementary Table 1), which was translated and culturally adapted into Brazilian Portuguese (as described below); and the Pain Catastrophizing Scale (PCS), which was used to assess the evidence of validity based on the relation to another variable of CQ. The responses to the items of both scales were treated as observed (manifest) variables, and their underlying constructs were modeled as latent variables.

**Table 1:** Distribution of participants (n=334) by sample characteristics, presented as n (%; 95% confidence interval ).

Characteristic	n (% ; 95% CI)
Sex	
Female	249 (74.6; 69.6-78.9)
Marital status	
Single	208 (62.3; 57.0-67.3)
Married / Common-law marriage	101 (30.2; 25.6-35.4)
Divorced / Separated	24 (7.2; 4.9-10.5)
Widowed	1 (0.3; 0.1-1.7)
Educational level	
Incomplete elementary education	2 (0.6; 0.2-2.2)
Completed elementary education	18 (5.4; 3.4-8.4)
Completed high school	199 (59.6; 54.2-64.7)
Completed higher education	115 (34.4; 29.5-39.7)
Monthly household income (US$*)	
≤ 231.33	27 (8.1; 5.6-11.5)
231.34 to 369.53	36 (10.8; 7.9-14.6)
369.54 to 1,592.78	137 (41.1; 36.0-46.5)
1,592.96 to 2,077.28	55 (16.5; 12.9-20.9)
> 2,077.28	78 (23.5; 19.2-28.3)

*Estimated from the quotation of 07/06/2025 of the Central Bank of Brazil (US $1.00 = R$5.42).

The CQ is a self-report psychometric scale developed to assess catastrophizing traits in individuals, regardless of the presence of pain.^2^ It consists of 24 items, grouped in one single factor, in which respondents indicate how frequently each statement applied to them over the past two weeks. Responses are given on a 5-point Likert-type response scale: 1 = Never, 2 = Rarely, 3 = Sometimes, 4 = Often, and 5 = Always. The PCS was administered in its Brazilian Portuguese adapted version.^5^ ^,^ ^20^ It includes 13 items, distributed across three dimensions that reflect the main cognitive components of the construct: rumination, magnification, and helplessness. Participants respond to the items using a five-point Likert-type response scale ranging from 0 (“never”) to 4 (“always”), indicating how often they experience certain pain-related thoughts.^21^

### TRANSLATION AND CULTURAL ADAPTATION OF THE CATASTROPHIZING QUESTIONNAIRE (CQ) INTO BRAZILIAN PORTUGUESE

The translation of the original English version of the Catastrophizing Questionnaire (CQ) followed established guidelines for the cultural adaptation of psychometric instruments.^16^ ^,^ ^22^ Initially, three independent translators, all native Brazilian Portuguese speakers with proven fluency in English, translated the scale. The translations were compared by two researchers from the present study, who prepared a reconciled version, which was considered the preliminary Portuguese version of the CQ. This preliminary version was then back translated into English by two independent translators. The researchers compared the original, the preliminary Portuguese version, and the back-translated version, confirming conceptual, idiomatic, and semantic equivalence. These findings suggest that the preliminary version is adequate for use within the Brazilian cultural context.

A pilot study was conducted with a sample from the target population using the preliminary Portuguese version of the CQ to assess item clarity. The Incomprehension Index (II) was calculated to determine the percentage of participants who reported difficulty understanding each item.^12^ An II below 15% is considered acceptable, indicating adequate clarity and cultural adaptation of the translated content.

The pilot study included 39 orthodontic patients, of whom 79.41% (n = 31) were female. The mean age of participants was 33.95 years (SD = 10.50). None of the items presented II above 15%, indicating good comprehension and acceptability of the Brazilian Portuguese version. Therefore, the preliminary version was adopted as the final version of the CQ in Portuguese. This version is presented in Supplementary Table 1, alongside the original English version of the CQ.

### PSYCHOMETRIC PROPERTIES OF THE BRAZILIAN PORTUGUESE VERSION OF THE CQ

The validity and reliability of the data obtained using the Brazilian Portuguese version of the CQ were assessed through internal structure validity, evidence of validity based on the relationship with another variable, and reliability coefficients.^23^

### EVIDENCE OF VALIDITY BASED ON INTERNAL STRUCTURE

Evidence of validity based on internal structure was assessed through factorial and convergent validity. Initially, the psychometric sensitivity of the items in the Portuguese version of the CQ was evaluated using descriptive statistics of item responses. Given the large sample size of the study, distribution of the responses to the items was assessed based on the absolute values of skewness and kurtosis, rather than formal normality tests, which are overly sensitive in large samples.^9^ Absolute values of skewness and kurtosis below 3 and 10, respectively, were considered indicative of no severe violation of normal distribution assumptions, thereby supporting the psychometric sensitivity of the items.^9^

Factorial validity was assessed through Confirmatory Factor Analysis (CFA) using the robust estimation method Weighted Least Squares Mean and Variance Adjusted (WLSMV). Model fit was evaluated using the Comparative Fit Index (CFI), Tucker-Lewis Index (TLI), Root Mean Square Error of Approximation (RMSEA), and Standardized Root Mean Square Residual (SRMR). Factor loadings (λ) were also examined. Adequate model fit was indicated by CFI and TLI values greater than 0.90, RMSEA below 0.10, SRMR below 0.08, and factor loadings (λ) greater than 0.50.^9^ If necessary, modification indices estimated via Lagrange Multipliers (LM) were examined to identify potential residual correlations between item errors.

Convergent validity was assessed through the Average Variance Extracted (AVE). AVE values greater than 0.50 were considered indicative of adequate convergent validity.^24^ The suggestion of unidimensionality of the factor model was evaluated using the following indices: Unidimensional Congruence (UniCo), Explained Common Variance (ECV), and Mean of Item Residual Absolute Loadings (MIREAL). In addition to the point estimates of these indices, their 95% confidence intervals were calculated using the bootstrap sampling method. UniCo values > 0.95, ECV > 0.85, and MIREAL < 0.30 suggest that the items can be treated as representing a single dimension.^25^

### RELIABILITY

The reliability of the data was assessed through internal consistency, estimated using the ordinal alpha (α), omega (ω), and composite reliability (CR) coefficients. Values of α, ω, and CR above 0.70 were considered indicative of adequate reliability.^18^ ^,^ ^24^

### EVIDENCE OF VALIDITY BASED ON RELATION TO ANOTHER VARIABLE

Initially, the fit of the first-order factor model (three factors: magnification, rumination, and helplessness) and the second-order hierarchical model (SOHM: second-order factor-catastrophizing) of the PCS was assessed. All items from this scale demonstrated adequate psychometric sensitivity (Supplementary Table 2, Sk ≤ |1.2|, Ku ≤ |1.3|). The PCS factor models presented an adequate fit to the data (CFI = 0.97, TLI = 0.96, RMSEA = 0.11, SRMR = 0.05, λ = 0.74-0.93, AVE = 0.71, α ≥ 0.88, ω ≥ 0.85, CR ≥ 0.88).

**Table 2:** Descriptive statistics of mean scores and participants’ responses to the items of the Portuguese version of the Catastrophizing Questionnaire (CQ ).

Item	Mean	Median	Standard Deviation	Minimum	Maximum	Skewness	Kurtosis
CQ1	2.6	3.0	1.2	1.0	5.0	0.32	-0.72
CQ2	3.3	3.0	1.1	1.0	5.0	-0.11	-0.71
CQ3	2.4	2.0	1.1	1.0	5.0	0.41	-0.55
CQ4	2.7	3.0	1.1	1.0	5.0	0.36	-0.43
CQ5	3.2	3.0	1.3	1.0	5.0	-0.11	-1.14
CQ6	2.5	2.0	1.3	1.0	5.0	0.51	-0.84
CQ7	2.2	2.0	1.1	1.0	5.0	0.72	-0.18
CQ8	2.2	2.0	1.1	1.0	5.0	0.61	-0.26
CQ9	2.9	3.0	1.3	1.0	5.0	0.13	-0.99
CQ10	2.3	2.0	1.2	1.0	5.0	0.68	-0.26
CQ11	2.2	2.0	1.2	1.0	5.0	0.87	-0.07
CQ12	1.7	1.0	1.0	1.0	5.0	1.51	1.69
CQ13	2.2	2.0	1.2	1.0	5.0	0.84	-0.28
CQ14	2.3	2.0	1.1	1.0	5.0	0.60	-0.43
CQ15	2.1	2.0	1.1	1.0	5.0	1.01	0.51
CQ16	2.0	2.0	1.0	1.0	5.0	0.91	0.38
CQ17	2.3	2.0	1.2	1.0	5.0	0.75	-0.20
CQ18	2.7	3.0	1.2	1.0	5.0	0.30	-0.84
CQ19	2.5	3.0	1.1	1.0	5.0	0.31	-0.65
CQ20	2.2	2.0	1.2	1.0	5.0	0.77	-0.38
CQ21	2.4	2.0	1.1	1.0	5.0	0.51	-0.47
CQ22	1.9	2.0	1.0	1.0	5.0	1.26	1.28
CQ23	3.2	3.0	1.2	1.0	5.0	-0.15	-0.75
CQ24	2.6	3.0	1.2	1.0	5.0	0.29	-0.80
Mean score	2.4	2.4	0.7	1.0	4.8	0.36	-0.22
Sum score	58.3	57.0	16.7	24.0	116	0.36	-0.22

The evidence of validity of the Portuguese version of the CQ, based on relation to another measure, was assessed through correlation analysis (r) between the CQ factor and both the first- and second-order factors of the PCS. A strong and positive correlation between the scales’ factors was expected (positive convergent validity).

## RESULTS

Table S1 presents the original English version of the CQ developed by Pike et al.,^2^ alongside the final version translated and culturally adapted into Brazilian Portuguese. 

### STUDY PARTICIPANTS

The study sample consisted of 334 patients undergoing orthodontic treatment with a mean age of 30.6 years (standard deviation = 12.4; 95% confidence interval = 29.3-32.0; median = 25.0; range = 18-70). Table 1 presents the other characteristics of the participants. 

### PSYCHOMETRIC PROPERTIES

#### Evidence of validity based on internal structure

Table 2 presents the descriptive statistics for item responses on the Portuguese version of the CQ. None of the items showed severe deviations from normal distribution, as indicated by skewness and kurtosis values within the recommended limits (|Sk|≤1.51 and |Ku|≤1.69), confirming the psychometric sensitivity of all items. The fit of the CQ factor model to the data (CFA) is shown in Table 3. The model demonstrated adequate overall fit; however, two items - Item 6 (“I think that we are facing a major environmental disaster that humankind will not survive,” λ = 0.34) and Item 19 (“I think that we will see another world war in the next few years,” λ = 0.31) - exhibited low factor loadings.

**Table 3:** Fit indices of the factor model of the Portuguese version of the CQ applied to Brazilian orthodontic patients.

		CFA								
Factor model	Refinement	CFI	TLI	RMSEA	SRMR	λ	AVE	α	ω	CC
Full model^a^	-	0.95	0.94	0.07	0.06	0.31-0.81	0.40	0.94	0.93	0.94
Refined 1	Correlation between errors of item 6 and item 19 (r = 0.46)	0.96	0.95	0.06	0.06	0.29-0.81	0.41	0.93	0.92	0.94
Refined 2	Exclusion of items 6 and 19	0.96	0.95	0.06	0.05	0.43-0.81	0.43	0.94	0.93	0.94

CFA: Confirmatory Factor Analysis; CFI: Comparative Fit Index; TLI: Tucker-Lewis Index; RMSEA: Root Mean Square Error of Approximation; SRMR: Standardized Root Mean Square Residual; λ: Factor loading (values represent the smallest and largest factor loadings observed across items); AVE: Average Variance Extracted; α: Ordinal alpha coefficient; ω: Omega coefficient; CR: Composite reliability. ^a^ The lowest factor loadings were observed for items 6 (λ = 0.34) and 19 (λ = 0.31), and the highest modification index (Lagrange Multiplier [LM]) was found between the residuals of these items (LM = 81.53). The full model was considered the most appropriate for the sample data.

Upon inspection of the modification indices, the highest value was observed between these two items (LM = 81.53). Consequently, two refinements of the factor model were tested: the addition of a correlation between the error of the Items 6 and 19 (Table 3 - Refined Model 1), and the exclusion of both items (Table 3- Refined Model 2). As shown in Table 3, neither refinement resulted in a substantial improvement in model fit. Although Items 6 and 19 contribute only modestly to the measurement of catastrophizing in this Brazilian orthodontic patient sample, they do not negatively affect the construct assessment. Therefore, to preserve the original structure of the CQ (including all 24 items), the full factor model was deemed the most appropriate for the Portuguese version applied to the present sample. Thus, the full model was retained for all subsequent analyses.

The Average Variance Extracted (AVE) was 0.40 (Table 3), which is below the recommended threshold of 0.50. This result appears to reflect the low factor loadings of some items, including Items 6 and 19. Therefore, when the AVE is interpreted alongside the adequate reliability coefficients (presented below) and the acceptable global model fit, the convergent validity of the CQ was considered acceptable. The indices of suggestion of unidimensionality were as follows: UniCo = 0.94 (95% CI: 0.93-0.98), ECV = 0.87 (95% CI: 0.86-0.90), and MIREAL = 0.14 (95% CI: 0.13-0.14). Both the point estimates and confidence intervals for ECV and MIREAL were within the reference values supporting unidimensionality, while UniCo had a borderline point estimate and a confidence interval that crossed its recommended threshold. Taken together, these indicators support and confirm the unidimensional structure of the CQ.

#### Reliability

All internal consistency indices exceeded 0.93 for the full factor model (Table 3), indicating adequate reliability of the data obtained using the Portuguese version of the CQ.

#### Evidence of validity based on relation to another variable

All correlations between the CQ factor and the PCS factors were positive, strong, and statistically significant (p < 0.001), confirming positive convergent validity (CQ vs. magnification: r = 0.62; CQ vs. rumination: r = 0.71; CQ vs. helplessness: r = 0.59; CQ vs. catastrophizing [PCS SOHM]: r = 0.68).

In summary, although two items showed low factor loadings and the AVE fell below the ideal threshold, the overall model fit, adequate reliability coefficients, and strong correlations with the PCS support the psychometric adequacy of the CQ in this sample. These findings attest to the validity and reliability of the data obtained to measure catastrophizing in the present study population.

## DISCUSSION

The present study translated and culturally adapted the Catastrophizing Questionnaire (CQ) into Portuguese. It also attested the psychometric properties of this version when applied to Brazilian orthodontic patients. The rationale for conducting this study is that the CQ, unlike other tools such as PCS, is a scale developed to assess catastrophizing independently of a pain context. This characteristic makes it particularly relevant in both clinical and research settings, as it allows for the identification of the respondent’s general tendency to catastrophize, regardless of a specific condition. 

The study indicated that participants from the target population adequately comprehended the content of the items in the Portuguese version of the CQ. This version showed satisfactory psychometric properties. However, two items exhibited low local fit (i.e., low factor loadings - λ) and a high residual correlation between them: Item 6 (“I think that we are facing a major environmental disaster that humankind will not survive”) and Item 19 (“I think that we will see another world war in the next few years”). Some cultural specificities of the Brazilian population may help explain these findings. Both items refer to scenarios of extreme severity. Although plausible in recent global contexts, such scenarios may be more distant from the historical and collective experience of Brazilians. In contemporary collective memory, Brazil is often perceived as having had limited direct involvement in the world wars and not experiencing those conflicts on its territory. In addition, Brazil has not experienced large-scale environmental disasters. These factors may reduce the extent to which these items effectively reflect the catastrophizing construct among Brazilian respondents.

In light of these findings, two refined factor models were tested to explore potential improvements in model fit. The first included a correlation between the errors of these items, while the second evaluated the exclusion of these items. Allowing correlations between errors that are not theoretically specified in the original model may introduce bias into parameter estimates.^26^ Item exclusion may reduce the conceptual coverage of the measured construct.^9^ Those strategies can compromise the interpretability of the findings. In the present study, we retained the full factor model without correlations or excluded items as the most appropriate solution. The decision was based on the conceptual relevance of both items in the current global context, and on the marginal improvement in model fit indices obtained by correlating the errors. In addition to demonstrating satisfactory fit, our decision enhances the comparability of our findings with future studies using the CQ.

These considerations highlight a broader issue in research using psychometric scales: the assumption that a scale will function equivalently across different populations, sociocultural contexts, and methodological procedures. However, not all psychometric scales maintain structural equivalence in culturally distinct settings. Administration methods (paper-based vs. digital) and recruitment strategies can influence the response patterns, even when the scale structure remains unchanged.^27^ Campos et al.^28^ compared self-image between Brazilian and Finnish populations using psychometric scales. They observed that while some measures demonstrated configural and measurement invariance, others did not. Such results highlight the importance of evaluating how latent constructs are perceived and measured across populations, especially when adapting scales for cross-cultural use. 

While the CQ was translated into Portuguese, it was culturally adapted for the Brazilian adult population. Thus, its use in other Portuguese-speaking countries should be approached with caution. These countries are connected by the Portuguese Language Orthographic Agreement,^29^ which promotes a largely unified written standard across Lusophone nations. Nevertheless, historical, sociocultural, and contextual differences may affect how item content and idiomatic nuances are interpreted.^5^ ^,^ ^17^ ^,^ ^22^ Before applying this version in other Lusophone populations, expert review and pilot testing are recommended to assess the need for further cultural adaptation. Subsequent psychometric evaluation, including the assessment of measurement invariance, to support score interpretation across countries. 

The Average Variance Extracted (AVE) was 0.40, which is below the recommended threshold of 0.50,^24^ indicating a limitation in convergent validity. This suggests that some items had low loading on the latent factor. However, Fornell and Larcker^24^ pointed out that AVE is a conservative estimate. Convergent validity may still be acceptable when composite reliability (CR) is adequate.^24^ In the present study, the remaining psychometric indicators, including global fit indices, CR, and other reliability estimates, were satisfactory. Furthermore, indices suggesting unidimensionality were estimated,^25^ and their values support the retention of the one-factor model.

Evidence of validity based on relations to other measures was supported by strong and significant correlations between the CQ factor and both first- and second-order factors of the PCS.^5^ ^,^ ^20^ ^,^ ^21^ It is important to emphasize that, unlike the PCS, which is designed for a pain-related context and is applied to individuals with current or past pain experiences, the CQ may offer an identification of catastrophizing traits independent of pain. This conceptual distinction is particularly relevant in orthodontic practice, where painful procedures can be anticipated and managed preventively within a patient-centered approach. The cognitive anticipation of painful events can shape the subjective experience of pain.^30^ ^,^ ^31^ Accordingly, the CQ may allow orthodontists to identify patients who are more prone to catastrophizing before appliance placement. 

Sociodemographic characteristics are also linked to psychosocial vulnerability, potentially shaping how patients experience and respond to treatment.^13^ Catastrophizing-related vulnerability has been reported to be associated with female sex, younger age, and disadvantaged social conditions.^20^ ^,^ ^32^ Although the present study was not designed to test sociodemographic categories as predictors, these variables may be useful for characterizing patient profiles and should be investigated in future studies. These considerations may help guide clinical attention for individuals with higher catastrophizing levels. This may include enhanced anticipatory guidance regarding the treatment experience, closer follow-up, and tailored orthodontic pain-management counseling.^3^ ^,^ ^6^ ^,^ ^33^ ^,^ ^34^

The total CQ score was originally conceived as the sum of responses to the 24 items, generating total scores from 24 to 120. While summing item responses is common, it assumes that all items contribute equally to the construct. This approach does not account for differences in factor loadings or the theoretical relevance of each item. As previously discussed, item exclusion is sometimes necessary to obtain adequate model fit to sample data. However, excluding items alters the sum score range, compromising interpretability and comparability across studies. Calculating the mean score instead standardizes the metric, but still assumes equal item weighting, which is unrealistic. This uniform weighting may compromise the validity of inferences drawn from the total score. Campos et al.^15^ emphasized that relying solely on total scores derived from summing or averaging disregards the underlying measurement model of the scale, including its factorial structure and potential differential item functioning. As an alternative, approaches that apply weights derived from factor analysis or models based on Item Response Theory (IRT) offer more accurate estimations of the construct. Future studies using the CQ are encouraged to explore these advanced scoring methods to reduce bias and obtain more accurate and representative total scores. 

The cross-sectional design and non-probabilistic sampling are limitations of this study. These aspects do not allow for longitudinal inferences or the assessment of temporal stability and treatment responsiveness. Although the use of convenience samples may hinder the generalizability of the findings, this approach is widely employed in studies aimed at scale adaptation and estimation of psychometric properties.^2^ ^,^ ^4^ ^,^ ^5^ ^,^ ^15^ ^,^ ^16^ ^,^ ^20^ ^,^ ^21^ ^,^ ^27^ Additionally, the study sample comprised orthodontic patients with relatively homogeneous profiles in terms of age, education, and access to healthcare services. Psychological variables that may be relevant to the catastrophizing construct were not assessed.

The study population was chosen to provide a clinically useful scale for assessing catastrophizing traits in orthodontic patients in the pre-treatment phase, that is, before appliance placement and the onset of orthodontic pain. In the future, it is expected that the CQ may contribute to identifying individuals with greater psychosocial vulnerability and support personalized strategies for managing pain and anxiety during orthodontic treatment. Additional studies in more diverse populational and clinical samples are needed to broaden the applicability and evaluate the psychometric properties of the Portuguese version of CQ across contexts.

Despite the limitations, the present study offers relevant contributions to multiple fields of knowledge. From a psychometric perspective, the study expands the potential uses of the CQ by demonstrating its applicability in a new population and context. Furthermore, the availability of a culturally adapted version for the Brazilian population represents a significant advancement, especially considering Brazil’s continental dimensions and its linguistic and cultural ties with other Portuguese-speaking countries. This may facilitate future adaptations with minimal modifications and support large-scale use of the scale in both clinical practice and research settings. 

## CONCLUSION

The Portuguese version of the CQ demonstrated adequate psychometric properties, indicating that it can be used to assess catastrophizing traits in Brazilian adults undergoing orthodontic treatment. Because the CQ measures this trait independently of a pain context, it is a unique and useful tool for identifying catastrophizing tendencies before appliance placement and the onset of orthodontic pain. In clinical practice, the CQ may support a patient-centered approach by screening individual psychosocial vulnerabilities and guiding tailored communication and behavioral strategies to improve treatment adherence. Future studies are encouraged to investigate its responsiveness and associations with sociodemographic variables in diverse populations. Overall, our findings highlight the CQ as a valuable addition to the orthodontic clinical toolkit that can provide valid and reliable data to inform personalized care.

## Data Availability

Due to the sensitive nature of the data and ethical restrictions, the datasets supporting this study are not publicly available.

## Appendix

**Supplementary Table 1:** Original version by Pike et al.* and final version translated into Portuguese of Catastrophizing Questionnaire ( CQ ).

Original version	Portuguese Version
Please indicate how often over the last two weeks the following statements have applied to you. Responses: 1- never, 2- rarely, 3- sometimes, 4- often, 5 always	Por favor, indique com qual frequência as seguintes afirmações se aplicaram a você nas últimas duas semanas. Respostas: 1- Nunca, 2- Raramente, 3- Às vezes, 4- Frequentemente 5- Sempre
1) If I have a problem, I wish somebody else would take the burden away from me.	1) Se tenho um problema, gostaria que outra pessoa tirasse esse peso de mim.
2) I think about all the ways that things can go wrong.	2) Eu penso em todas as maneiras que as coisas poderiam dar errado.
3) I imagine that I might have a serious health issue.	3) Eu imagino que posso ter algum problema sério de saúde.
4) I think about things that others would say are unlikely to happen.	4) Eu penso sobre coisas que os outros diriam ser improváveis de acontecer.
5) If I have an exam, I think that if I fail it will affect my whole future.	5) Se eu tenho que passar por alguma avaliação, penso que se eu falhar, isso afetará todo o meu futuro.
6) I think that we are facing a major environmental disaster that humankind will not survive.	6) Eu penso que estamos vivendo um grande desastre ambiental e que a humanidade não irá sobreviver.
7) I think that a disaster is going to happen to me.	7) Eu penso que algum desastre irá acontecer comigo.
8) If I have a disagreement with a person I care about, I think that we will not make up.	8) Se eu tenho um desentendimento com alguém que eu me importo, penso que não faremos as pazes.
9) I overthink and then become unable to decide what to do.	9) Eu penso demais sobre as coisas e acabo sem tomar decisões.
10) I think I am going to make a big mistake soon.	10) Eu acho que vou cometer algum grande erro em breve.
11) If I have a medical symptom (headache, heart palpitations, stomach ache), I think I must have a serious disease.	11) Se eu tenho algum sintoma físico (dor de cabeça, palpitações, dor no estômago), penso que é uma doença séria.
12) If I have an illness, I don't believe that treatment will work.	12) Se eu tenho uma doença, penso que o tratamento não vai funcionar.
13) If I text a friend and they don’t message me back, I immediately think that they're upset with me.	13) Se eu envio uma mensagem a um amigo e este(a) não responde, logo penso que esse amigo(a) está chateado(a) comigo.
14) I think that any problem will only get worse as time passes.	14) Eu acho que qualquer problema apenas irá piorar com o tempo.
15) If my partner is late home from work, I think that they have been in an accident.	15) Se meu parceiro(a) está atrasado do trabalho, penso que ele(a) se envolveu em algum acidente.
16) I think that what I am going through is much worse than what others have experienced.	16) Eu acho que o que estou passando é muito pior do que outros já passaram.
17) I think I am going to lose someone close to me forever.	17) Eu acho que vou perder alguém próximo de mim para sempre.
18) I think that I will always have money problems.	18) Eu acho que sempre vou ter problemas financeiros.
19) I think that we will see another world war in the next few years.	19) Eu acho que veremos outra guerra mundial nos próximos anos.
20) If I have a bad month at work, I think that I will get fired.	20) Eu penso que se tive um mês ruim no trabalho, serei demitido(a).
21) I think that the worst-case scenarios are very likely to happen.	21) Eu acho que as piores situações possíveis são bem prováveis de acontecer.
22) I think that my house will be burgled.	22) Eu acho que minha casa será assaltada.
23) I think about what will happen if I make a mistake.	23) Eu penso no que irá acontecer se eu cometer algum erro.
24) I think that I am not very good at finding ways to solve my problems.	24) Eu acho que não sou muito bom em encontrar maneiras de resolver os meus problemas.

**Supplementary Table 2:** Descriptive statistics of participants’ responses to the items of the Brazilian Portuguese version of the Pain Catastrophizing Scale ( PCS ).

Item	Mean	Median	Standard Deviation	Minimum	Maximum	Skewness	Kurtosis
PSC1	1.5	1	1.1	0	4	0.45	-0.45
PSC2	1.0	1	1.0	0	4	1.04	0.73
PSC3	0.9	1	1.0	0	4	1.19	1.04
PSC4	0.9	1	1.1	0	4	1.13	0.75
PSC5	0.9	1	1.0	0	4	1.04	0.50
PSC6	1.4	1	1.2	0	4	0.47	-0.57
PSC7	1.0	1	1.1	0	4	0.95	0.24
PSC8	2.1	2	1.4	0	4	0.02	-1.26
PSC9	1.4	1	1.2	0	4	0.65	-0.34
PSC10	1.4	1	1.2	0	4	0.71	-0.21
PSC11	1.9	2	1.3	0	4	0.21	-1.04
PSC12	1.1	1	1.1	0	4	0.89	0.20
PSC13	1.3	1	1.2	0	4	0.76	-0.36
